# Assessing the effects of free fall conditions on damage to chickpea seeds: A comprehensive examination of seed deterioration

**DOI:** 10.1002/fsn3.4041

**Published:** 2024-02-20

**Authors:** Farzad Delfan, Feizollah Shahbazi, Hamid Reza Eisvand, Saba Shahbazi

**Affiliations:** ^1^ Faculty of Agriculture Lorestan University Khoramabbad Iran; ^2^ Bushehr University of Medical Sciences Bushehr Iran

**Keywords:** chickpea, free fall, impact surface, mechanical damage, seed moisture

## Abstract

Impact damage is the most destructive effect on the seeds during harvesting, handling, and storage, both on‐farm and off‐farm. The chickpea seeds' dicotyledonous characteristics and large mass and size make them susceptible to mechanical damage under impact loading. Tests were conducted to determine the extent of damage to chickpea seeds due to the impact caused by free fall. The extent of internal damage to the chickpea seeds was determined, which included the measurement of seed deterioration by the accelerated aging method (percentage loss in germination in the accelerated aging test) and the measurement of electrical conductivity. Three independent variables were used in the test, namely: (a) drop height (3, 6, 9, and 12 m), (b) impact surface (concrete, metal, plywood and seeds on seeds), and (c) seed moisture content (10%, 15%, 20%, and 25% w.b). The results showed that drop height, impact surface, and moisture content had significant effects (*p* < .01) on the loss in germination percentage and change in electrical conductivity of chickpea seeds. In terms of loss in germination, the highest damage to seeds occurred at the metal impact surface (41.96%) and the least at the seed on the seed (29.71%). The highest amount of electrical conductivity was related to the seeds dropped on the metal (36.09 μS cm^−1^ g^−1^) and the lowest was related to seed‐on‐seed contact (21.68 μS cm^−1^ g^−1^). By increasing the drop height from 3 to 12 m, the loss in germination and electrical conductivity of seeds increased from 27.74% to 48.08% and from 18.72 to 40.47 μS cm^−1^ g^−1^, respectively. Increasing the moisture content of chickpea seeds from 10 to 25% causes a decrease in the amount of damage to the seeds in terms of electrical conductivity (from 38.40 to 21.18 μS cm^−1^ g^−1^), but increases the damage in the form of a loss in the percentage germination in the accelerated aging test (from 29.22% to 42.88%). To reduce the impact damage to peas caused by free fall, the height of the fall should be limited to about 6 m, and they should be prevented from hitting hard and rough surfaces.

## Graphical abstract

The drop height was a significant factor in causing damage to the chickpea seeds. Increasing the drop height caused more damage. On striking a surface at speeds above 7 m s^−1^, internal damage to chickpea seeds had the highest values. To reduce impact damage to chickpeas caused by free fall, the drop height should be limited to about 6 m. As the moisture level increased, the electrical conductivity of chickpea seeds decreased; however, the physiological damage (loss in germination percentage in the accelerated aging test) to the seeds increased.

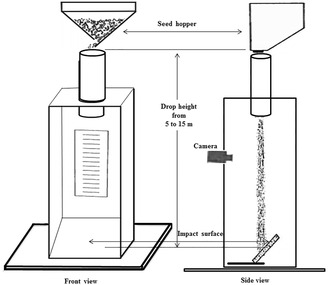

## 1 INTRODUCTION

With the increasing global population, reducing food losses has become crucial for ensuring food security worldwide. Among the various potential hazards that can harm the quality and viability of crops, mechanical damage during harvesting and handling is a significant concern (Shahbazi et al., 2023). Consequently, the evaluation and modeling of mechanical damage for grains and oilseeds have garnered significant attention in recent years (Chen et al., 2020; Nadimi et al., 2022; Nadimi, Divyanth, & Paliwal, 2023; Su et al., 2019a, 2019b).

Chickpea (*Cicer arietinum* L.) is an important legume that plays a significant role in providing the necessary protein for individuals in developing countries (Shahbazi, 2011). However, due to the large size and mass of chickpea seeds, they are highly susceptible to mechanical damage from impact loads (Delfan et al., 2023). Additionally, the germ tip is located in the protruding structure, which is a characteristic of dicotyledons (Shahbazi, 2011).

The primary cause of chickpea losses in its supply chain is the impact stress caused by various factors, including free fall during harvesting, handling, and post‐harvest processing. Seeds may fall from different heights and collide with various surfaces during these operations, resulting in impact stress that leads to losses in both quality and quantity. Mechanical damage caused by impact force can be classified as external damage and internal damage (Li et al., 2022; Paulsen et al., 2019; Shahbazi et al., 2023). External damage includes visible fissures, cracks, and fractures on the seed's surface. On the other hand, internal damage, also known as “stress cracking,” refers to fissures or small cracks in the seed embryo caused by tensile or compressive stress during or after drying, rehydration, or impact loading (Shahbazi & Shahbazi, 2023).

Seeds with high‐stress cracks are prone to breakage during subsequent unit operations such as processing and handling. Additionally, seeds with stress cracks are vulnerable to insect and mold infestation during storage and typically have low viability (Rybchynskyi, 2020). In general, mechanical damage to seeds can result in a decrease in their economic value, reduced storage potential (Olisa et al., 2021), health issues (Deng et al., 2021; Fan et al., 2017; Narendran et al., 2019), and increased downstream costs associated with processing and product manufacturing.

During the harvesting, handling, and transportation processes, seeds undergo free fall and sustain impact damage in combines, railcars/trucks, augers, and conveyors composed of different materials (Shahbazi et al., 2023). The extent of impact damage is influenced by several factors, including the height of the drop, characteristics and conditions of the seed/grain (density, size, moisture, and temperature), ambient temperature, the material of the impact surface, and the angle of collision between the seeds and the surface (Chen et al., 2020; Shahbazi, 2021; Shahbazi et al., 2023).

Researchers have analyzed drop tests of corn, soybean, and wheat seeds on various contact surfaces in multiple studies (Bartkowiak et al., 2019; Li et al., 2020; Shah et al., 2001; Shahbazi & Shahbazi, 2022a, 2022b, 2023). The results of these studies consistently show that the damage to seeds caused by free fall increases with the height of the fall but may decrease with moisture content and temperature. Tang et al. (1991) observed that lentil seeds were more likely to break when subjected to free fall at −25°C compared to a room temperature of 22°C. In another relevant study, Shahbazi and Shahbazi (2022a, 2022b) investigated the effects of cushion boxes and closed let‐down leaders on reducing damage to corn seeds caused by free fall. They considered different seed moisture levels and drop heights and found that implementing such systems could decrease damage parameters, such as breakage percentage and cracking index.

Contributing to the assessment of chickpea seed damage caused by free fall under different test conditions, this study aimed to: (a) evaluate the physiological damage to chickpea seeds caused by impact from free fall at various heights, different seed moisture levels, and different impact surfaces; (b) determine the reduction in storage potential of chickpea seeds, such as germination percentage loss and change in electrical conductivity, due to impact from free fall at different heights, sample moisture levels, and impact surfaces; (c) measure the mean values of seed velocities, including single velocity and mass flow velocity, when dropped from different heights; and (d) recommend a safer drop height for the design and operation of handling equipment.

## 2 MATERIALS AND METHODS

### 2.1 Sample

Azad variety chickpeas were manually harvested when they were fully ripe for free fall testing. The samples were then stored at a temperature of 5°C and a relative humidity of 90% until the experiments commenced (Delfan et al., 2023). The initial moisture content of the seeds was determined using the ASABE standard S352.2 (ASABE S352.2, 2006) and was found to be approximately 10%. To create seed samples with higher moisture levels, pre‐calculated quantities of distilled water were added, and these samples were subsequently kept at a temperature of 4°C for a duration of 10 days.

### 2.2 Free fall test

Laboratory experiments were carried out to simulate the free fall of chickpeas and evaluate the effects of dropping from different heights and contacting different surfaces at varying moisture contents of the seeds, as well as the resulting damage (Shahbazi et al., 2023). Four drop heights (3, 6, 9, and 12 m) were chosen to represent conditions that are commonly encountered in the field, at seed cleaning plants, and during processing at seed elevators. The contact surfaces included concrete, metal, plywood, and seed‐on‐seed interactions. All experiments were carried out in a temperature‐controlled building with an ambient temperature of 20°C.

**FIGURE 1:**
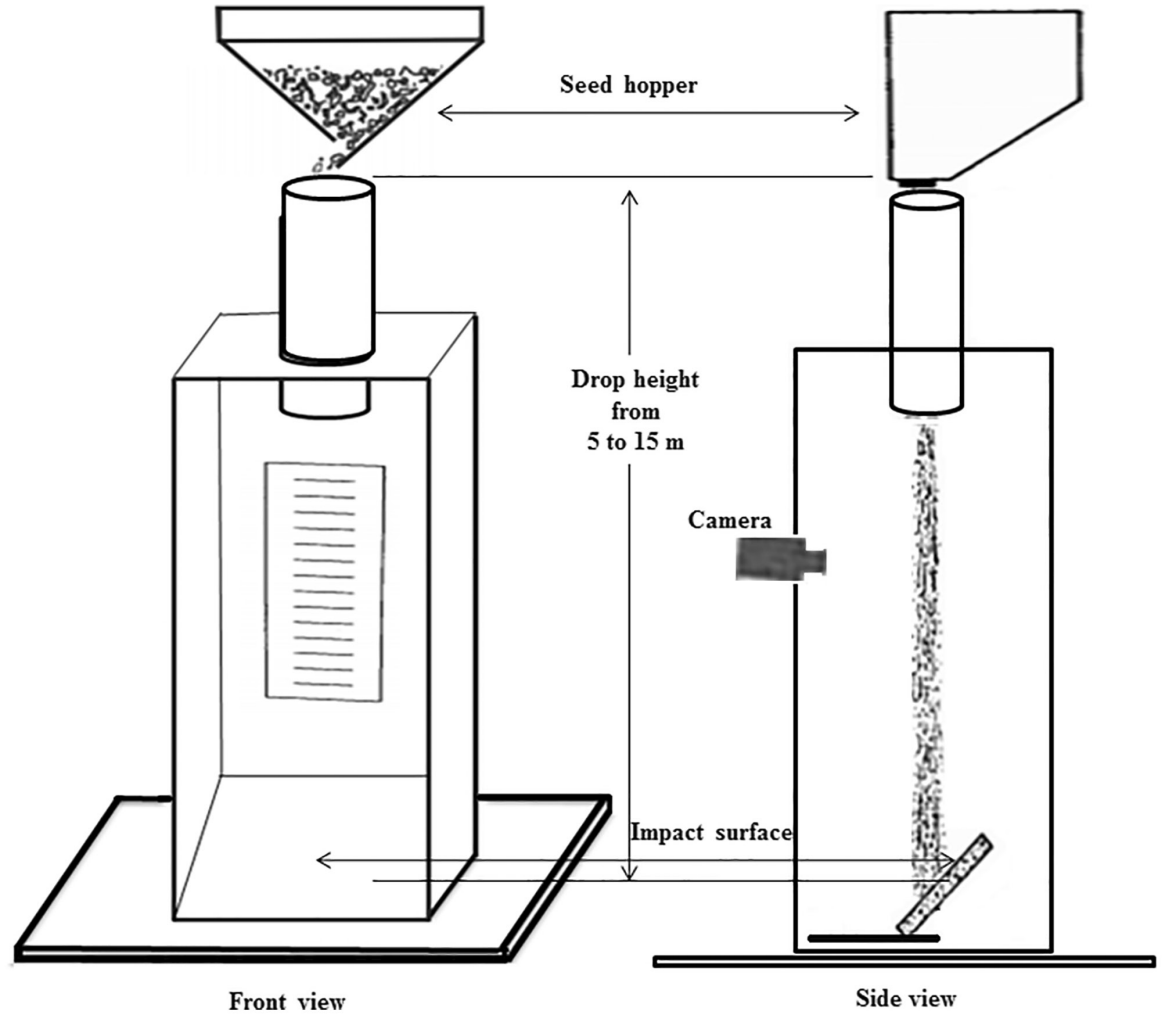
Diagarm of the drop test apparatus.

To simulate the free fall of chickpeas, PVC pipes with a 100 mm diameter were utilized to vary the drop heights. A 40‐mm opening funnel was affixed to the top of each tube. In each experimental run, 500 g of seed samples were released into the PVC tubes, with three replications conducted. The seeding flow rate was maintained at 0.25 kg s^−1^. The bottom of the tube led into a wooden chamber (Figure 1). To prevent seed jumping, a foam core was positioned in front of the chamber. The contact test surfaces, inclined at a 45° angle, were placed inside the wooden chamber to simulate seed dropping into an empty hopper bucket (Figure 1). These contact surfaces measured 500 mm and 400 mm in size. For seed‐on‐seed drop tests, a seed layer with the same moisture content as the tested seed sample was used as the test surface. A thin cellophane cover was delicately placed on the seed test surface to facilitate easy removal of the test samples while maintaining contact with the seed surface. Damage assessments were conducted after the drop tests.

### 2.3 Assessment of seed damage caused by a free fall

This study aimed to assess the internal damage to chickpea seeds resulting from free fall at various heights onto different impact surfaces and to evaluate the impact of free fall on seed storage potential. The extent of internal damage was measured and evaluated as an indication of seed deterioration through accelerated aging and changes in electrical conductivity. Damage measurement tests were conducted on intact seeds from each treatment.

For the accelerated aging test, 40 g of various seed samples (including control samples and free‐fall‐treated samples) were weighed and placed in an accelerated aging chamber at 42°C and 100% relative humidity for 72 h. Afterward, a standard germination test was conducted to determine the decrease in germination percentage compared to control samples (Olisa et al., 2021). The difference in germination percentage between control samples (without free fall) and samples subjected to different free fall tests was calculated as a loss in germination (Shahbazi & Shahbazi, 2022b).

The electrical conductivity test assesses the conductivity of electrolytes released from plant tissue, which indicates the weakness of the membrane structure and the poor condition of the seeds. When these seeds are soaked in water, the leakage of electrolytes, particularly amino and organic acids, increases the conductivity of the water. To conduct the electrical conductivity test, four samples of 50 seeds from each treatment were randomly selected, weighed, and placed in containers with 250 mL of distilled water (Kordi & Shahbazi, 2020). All containers were covered with aluminum foil and incubated at 20°C for 24 h. After the soaking period, the electrical conductivity of the solution was measured using an electrical conductivity meter (Jenway 4010; UK).

### 2.4 Measuring the chickpea seed's velocity during free fall

The velocity of seeds in free fall, both as individual seeds and in mass flow, was measured using a Nikon D53000 digital camera. The camera captured the seeds just before they hit the impact surfaces. To record the seed velocity as they exited the PVC pipe (Figure 1), a distance of around 1 m was maintained between the lower end of the pipe and the impact surface. To facilitate observation of the distance traveled by the seeds, lines were drawn at 5 cm intervals and placed in the background (Figure 1). Videos were recorded for both scenarios, single seed and mass flow, to determine the potential impact of air resistance on their velocity. Tracking the grains in the mass flow proved challenging, so the velocity of the grains was recorded at the start and end of the mass flow.

### 2.5 Experimental design and data collection

In this study, drop height (H) with three levels, impact surface (S) with four levels, and moisture content (M) with four levels were selected as dependent parameters, and the loss in germination percentage in accelerated aging tees and change in the electrical conductivity of seeds were considered as the dependent parameters. The factorial experimental design with three replications was adopted, and a total of 144 (3 × 4 × 4 × 3) sets of tests were conducted in the laboratory. The analysis of variance of the data for determining the significance of main and interaction effects was performed by using the Statistical Package in the Social Sciences (SPSS) for Windows version 19.0 (Gray & Kinner, 2012). Data were plotted, and analysis was carried out at *p* < .05 to compare the means of all possible pairs of treatments.

## 3 RESULTS AND DISCUSSION

In the laboratory, we measured the internal damages to chickpea seeds, which included the percentage loss in germination and the change in electrical conductivity. These damages were caused by the impact of free fall and were also affected by the independence of the seeds. To understand the impact of drop height (H), impact surface (S), and moisture content (M) on electrical conductivity and loss in germination during accelerated aging, we conducted an ANOVA in a factorial design. The results presented in Table 1 showed that the individual effects of drop height, contact surface, and seed moisture on the percentage loss in germination and electrical conductivity of chickpeas were significant at a 1% level. However, the effect of drop height on both types of damage was higher compared to the impact surface and seed moisture (as indicated by higher mean square values). This suggests that an increase in drop height has a greater impact energy than the impact surface and seed moisture. The ANOVA table also reveals that the interaction effect between impact surface and moisture on the percentage loss in germination was significant at the 1% probability level. Furthermore, the interaction effects of drop height and impact surface, drop height and moisture, and moisture and impact surface were all significant on the electrical conductivity of chickpea seeds. Moreover, the interaction effect of the three independent variables on the electrical conductivity of chickpea seeds was significant at a 1% level.

**TABLE 1:** Analysis of variances (mean square) for the effects of independent variables on dependent variables.

Independent variables	Degree of freedom	Dependent variable (mean square)	
		Electrical conductivity	Loss in the accelerated aging test germination
Drop height (H)	3	4117.52**	3724.98**
Impact surface (S)	3	1998.24**	1462.02**
H × S	9	25.08**	0.01^ns^
Moisture content (M)	3	2711.25**	1689.58**
H × M	9	36.54**	0.01^ns^
S × M	9	17.98*	32.34**
H × S × M	27	18.25**	0.01^ns^
Error	128	7.77	4.54

*Note*: **p < .01, *p < .05.

Abbreviation: ns, not significant.

The results of comparing the means for chickpea seed damage, such as reduced germination and increased electrical conductivity, caused by free fall under various test conditions are presented in Table 2. Table 2 indicates that the measured damage values were significantly influenced by independent variables, such as moisture content, contact surface, and drop height.

**TABLE 2:** Comparison of mean results for percentage germination loss and electrical conductivity of tested seeds at different levels of drop height, impact surface, and moisture content.

Variable	Depended on variables	
	Electrical conductivity (μS cm^−1^ g^−1^)	Loss in germination in the accelerated aging test (%)
Drop height (m)		
3	18.72d	27.74d
6	26.73c	32.52c
8	33.16b	39.08b
12	40.47a	48.08a
Impact surface		
Metal	36.09a	41.96a
Concrete	33.64b	40.34b
Wood	27.67c	35.41c
Seed on seed	21.68d	29.71d
Moisture content (%)		
10	38.40a	29.22d
15	33.01b	35.46c
20	26.49c	39.86b
25	21.18d	42.88a

*Note*: a–d: Columns with the same letter have mean values that are not significantly different (*p* < .05).

### 3.1 Effects of drop height

Consistent with the data in Table 2, an increase in drop height resulted in a higher percentage of germination loss for chickpeas. Additionally, the electrical conductivity of chickpea seeds increased with higher drop heights. In terms of the percentage loss in germination of chickpea seeds under various testing conditions, including different impact surfaces and moisture levels, the lowest mean value recorded was 27.74%. This value was observed at a dropping height of 3 m. At a dropping height of 6 m, the mean values of germination reduction increased to 32.52%, which is 1.17 times (17.23% more) higher than the value at 3 m. The deterioration of chickpeas due to the decrease in germination percentage at a dropping height of 9 m was 39.08%, representing a 40.87% increase compared to 3 m and a 20.17% increase compared to 6 m. The highest average loss in germination of seeds, at 48.08%, occurred at a drop height of 12 m, indicating severe damage to chickpea seeds with approximately 50% deterioration. Table 2 reveals a significant difference in the average percentage of germination losses at different drop heights (*p* < .05). The data on loss in germination percentage suggest a notable decline in the storage potential of chickpeas as the drop height increases.

During the accelerated aging test, healthy and undamaged seeds demonstrate better tolerance to harsh and unsuitable conditions of high temperatures and humidity, resulting in higher germination percentages compared to damaged seeds, as noted by other researchers (Rahman et al., 2004). Corn seeds subjected to an increased drop height from 5 to 15 m exhibited a 50% decrease in germination percentage during accelerated aging testing (Shahbazi & Shahbazi, 2022b). Seed deterioration is a dynamic process, with damaged areas gradually expanding to affect living embryonic tissues, leading to a decline in seed quality. Physical and physiological damage to the cell membrane is the primary cause of seed aging and deterioration, accompanied by enzymatic, respiratory, and hormonal changes, reduced protein production, genetic damage, and the accumulation of toxic metabolites (Shahbazi & Shahbazi, 2022b). Therefore, an increase in the drop height of chickpea seeds leads to greater damage, resulting in seed deterioration and reduced germination potential below the standard range (80%) required for seed certification. As a result, the decrease in germination potential due to free fall during handling can cause significant germination issues, ultimately reducing crop yield upon introduction into the field.

The results also showed that as the drop height increased, the average electrical conductivity of chickpea seeds increased (Table 2). The highest and lowest electrical conductivity values were 40.47 and 18.72 μS cm^−1^ g^−1^, respectively, corresponding to seeds dropped from heights of 12 and 3 m (Table 2). Additionally, seeds dropped from heights of 6 and 9 m had mid‐range electrical conductivity values of 26.73 and 33.16 μS cm^−1^ g^−1^, respectively. Furthermore, there were significant differences between the mean electrical conductivity values of seeds at different drop heights (*p* < .05). Measuring electrical conductivity is an important test for assessing seed viability. Mechanical damage to the seed coat and subsequent leakage of internal components result in higher electrical conductivity in damaged seeds. This finding is supported by previous studies (Rahman et al., 2004). According to the International Seed Testing Association (ISTA, 2009), seeds with an electrical conductivity below 25 μS cm^−1^ g^−1^ are considered to have strong and healthy structures. Seeds with electrical conductivity values between 25 and 29 μS cm^−1^ g^−1^ are suitable for early‐season planting, but they carry a risk of germination and weak plant establishment under unfavorable conditions. Seeds with electrical conductivity ranging from 30 to 43 μS cm^−1^ g^−1^ have relatively weak structures and are not recommended for early planting, especially in unfavorable conditions. Seeds with electrical conductivity values exceeding 43 μS cm^−1^ g^−1^ have weak structures and are unsuitable for planting (Sadeghi et al., 2013). Based on this classification, chickpea seeds dropped from heights greater than 9 m have electrical conductivity values exceeding 30 μS cm^−1^ g^−1^, indicating relatively weak structures and unsuitability for planting.

Increasing the drop height is predicted to result in higher impact energy and stress cracking on the seeds, leading to increased damage (Delfan et al., 2023; Shahbazi & Shahbazi, 2023). Seed coat cracking is a quality concern in legumes such as chickpeas as well, where it may impact seed quality, vigor, and germination percentage (Wang et al., 2024; Wang & Cichy, 2023). This adverse effect has been observed in previous studies on corn seeds (Shahbazi & Shahbazi, 2022a). Therefore, minimizing the drop height is necessary. One approach to achieving this is by utilizing systems that researchers have used (Shahbazi & Shahbazi, 2022a, 2022b) to reduce the velocity and drop height of seeds during loading or unloading in bins.

### 3.2 Effects of impact surface

The mean values of loss in germination percentage in the accelerated aging test and the change in electrical conductivity resulting from damage to chickpea seeds colliding with an impact surface were highest on metal, followed by concrete, plywood, and seed‐on‐seed (Table 2). This trend was previously reported by Evans et al. (1990) for soybeans. According to the data in Table 2, there are significant differences between the mean values of seed damage using different impact surfaces (*p* < .05). Under various experimental conditions, including different levels of drop height and moisture content, chickpea seeds that dropped on metal experienced a higher loss in germination percentage in the accelerated aging test at 41.96%. On the concrete surface, the percentage loss in germination was 40.34%, approximately 4% lower than that of metal. Seed samples that impacted the plywood surface had an average loss in germination percentage of 35.41%, about 15% lower than metal and about 4% lower than concrete. When seeds dropped on other seeds, a significantly lower average loss in germination of 29.71% was observed. This indicates that the seeds‐on‐seeds impact surface significantly reduced the induced mechanical damage to seeds by about 29% compared to metal, 26% compared to concrete, and 16% compared to plywood. In terms of electrical conductivity, chickpea seed samples that were dropped on a metal surface showed a higher average value of 36.09 μS cm^−1^ g^−1^. Similarly, the average values for seeds impacted on concrete, plywood, and other seeds were 33.64, 27.67, and 21.68 μS cm^−1^ g^−1^, respectively (Table 2). These findings suggest that seeds that come into contact with hard surfaces experience increased damage. This adverse effect has been observed in previous studies on corn seeds (Shahbazi et al., 2023). Therefore, it is crucial to prevent seed impacts on rough and hard surfaces during handling and processing. One potential solution is to shield these surfaces with soft and flexible materials, which can absorb impact energy and protect the seeds from impact damage (Shahbazi, 2021).

### 3.3 Effects of the moisture content

The data in Table 2 indicate that as the moisture content of seeds increased, there was an increase in the amount of damage, resulting in a loss in germination percentage; however, the amount of damage caused in terms of electrical conductivity decreased. This can be attributed to the fact that moisture affects the elastic properties of agricultural products, including seeds, causing different reactions in terms of damage types. Additionally, significant differences were observed in the mean values of both types of damage to chickpea seeds at various levels of moisture content (*p* < .05). Increasing the moisture content of the seeds from 10% to 25% resulted in an increase in the mean loss in germination percentage from 29.22% to 42.88%. The mid ranges of the loss in germination percentage were 35.46 and 39.86% for moisture contents of 15% and 20%, respectively. This finding suggests that chickpea seeds with lower moisture contents are more resilient to physiological damage (reduction in germination percentage) compared to those with higher moisture values, which aligns with previous studies (Khazaei et al., 2008; Shahbazi, 2021; Shahbazi et al., 2017). The higher moisture contents may have led to increased flexibility in the seed tissues, transferring impact energy to the embryo and further reducing the germination percentage, as reported by Khazaei et al. (2008) for wheat, Shahbazi et al. (2012) for pinto beans, and Gu et al. (2019) and Shahbazi and Shahbazi (2022b) for corn seeds. Higher seed moisture content can reduce mechanical seed coat damage at harvest (Uebersax et al., 2022).

The data in Table 2 show that as the moisture content of chickpea seeds decreased, the electrical conductivity increased. This observed trend appears reasonable because, at lower moisture contents, the seeds become more brittle and susceptible to mechanical damage, resulting in a higher rate of leakage and therefore a higher electrical conductivity. This is similar to what was reported by Olisa et al. (2021) for corn seeds. Under different impact surfaces and drop heights, at a moisture content of 10%, the seeds had a higher average electrical conductivity value of 38.40 μS cm^−1^ g^−1^, which is greater than the normal value of 25 μS cm^−1^ g^−1^ (ISTA, 2009). At 15% moisture content, the average electrical conductivity value was 33.01 μS cm^−1^ g^−1^, which was also higher than the normal value but approximately 14% lower than that of the 10% moisture content. For a moisture content of 20%, the average electrical conductivity value of the seeds was 26.49 μS cm^−1^ g^−1^, which fell somewhat within the normal range (25 μS cm^−1^ g^−1^) and was approximately 31% and 20% lower than those of the 10% and 25% moisture contents, respectively. At 25% moisture content, the least damage in terms of increased electrical conductivity was observed, with a mean value of 21.21 μS cm^−1^ g^−1^, which is lower than the optimal range of 25 μS cm^−1^ g^−1^.

These findings indicate that the moisture level of chickpea seeds is a crucial factor that impacts mechanical damage and, consequently, the quantity of wasted product. This consideration should be taken into account during harvesting and postharvest processing when impact damage is likely to occur. However, if the chickpea seeds are intended for planting or consumption, the situation is different. On the other hand, higher moisture content leads to a decrease in physiological damage, specifically seed deterioration. Nevertheless, it also results in a decrease in physical damage, such as internal cracks, leading to more material leakage. This highlights the necessity for further research on this matter, including the impact of damage on seed storage potential and changes in chemical properties like protein content and color at various moisture levels.

### 3.4 Interaction effect of impact surface and moisture content

**FIGURE 2:**
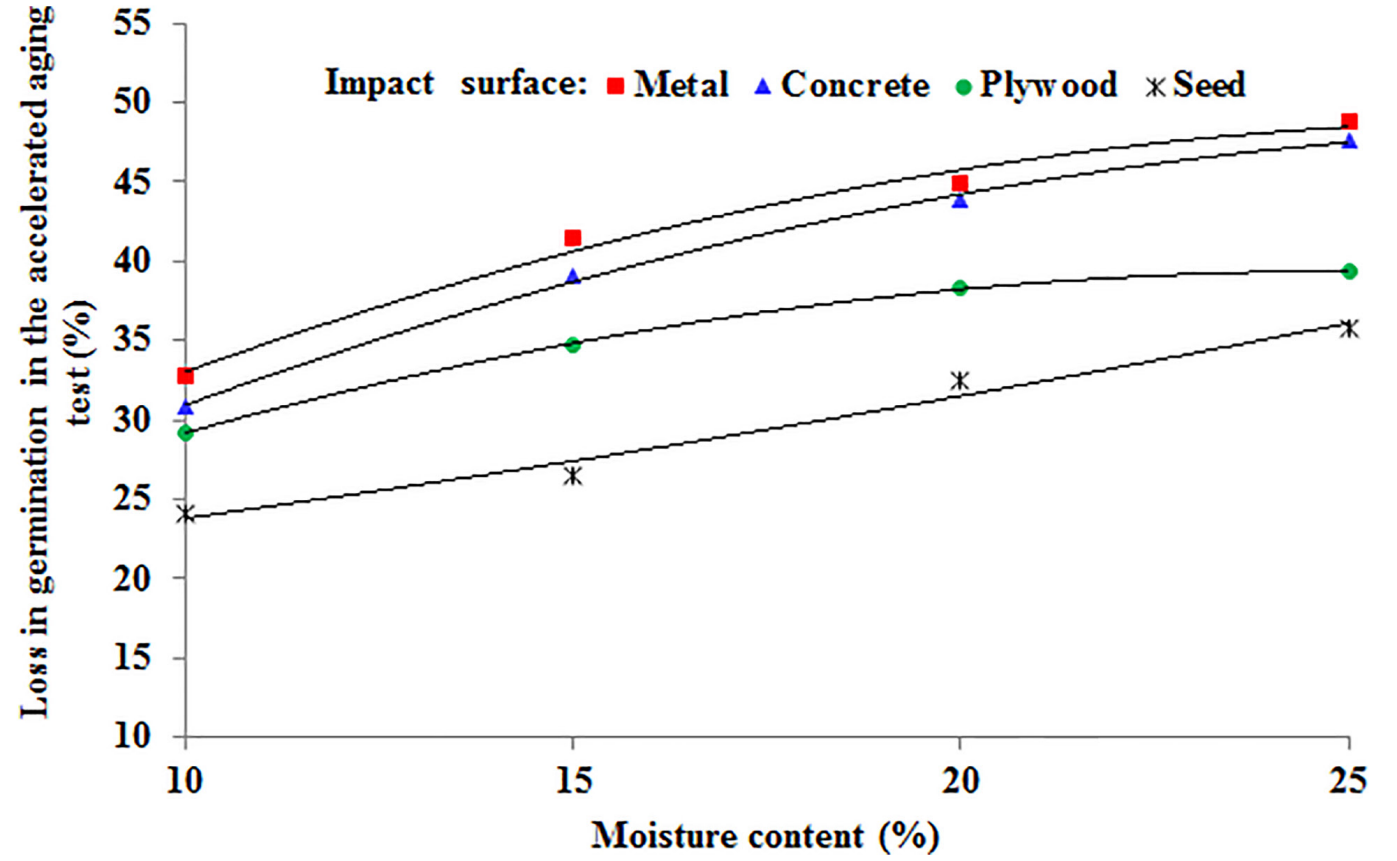
The loss in germination percentage of chickpea seeds in the accelerated aging test in the interaction effect of impact surface and moisture content.

In Figure 2, the interaction effect of impact surface and moisture content on the loss in germination percentage of chickpea seeds due to free fall is depicted. When seeds were dropped on seeds, the difference in the loss in germination was lower compared to seeds dropped on metal or concrete, particularly at higher moisture content (25%) compared to lower moisture (10%). The interaction effect of seed‐to‐seed impact surface and 10% moisture content resulted in the lowest value of the loss in the germination of chickpea seeds at 24.13% (the lowest moisture level). Conversely, the combination of seeds falling on the metal surface and 25% moisture (the highest level of moisture) created the highest loss in germination at 48.76%.

**FIGURE 3:**
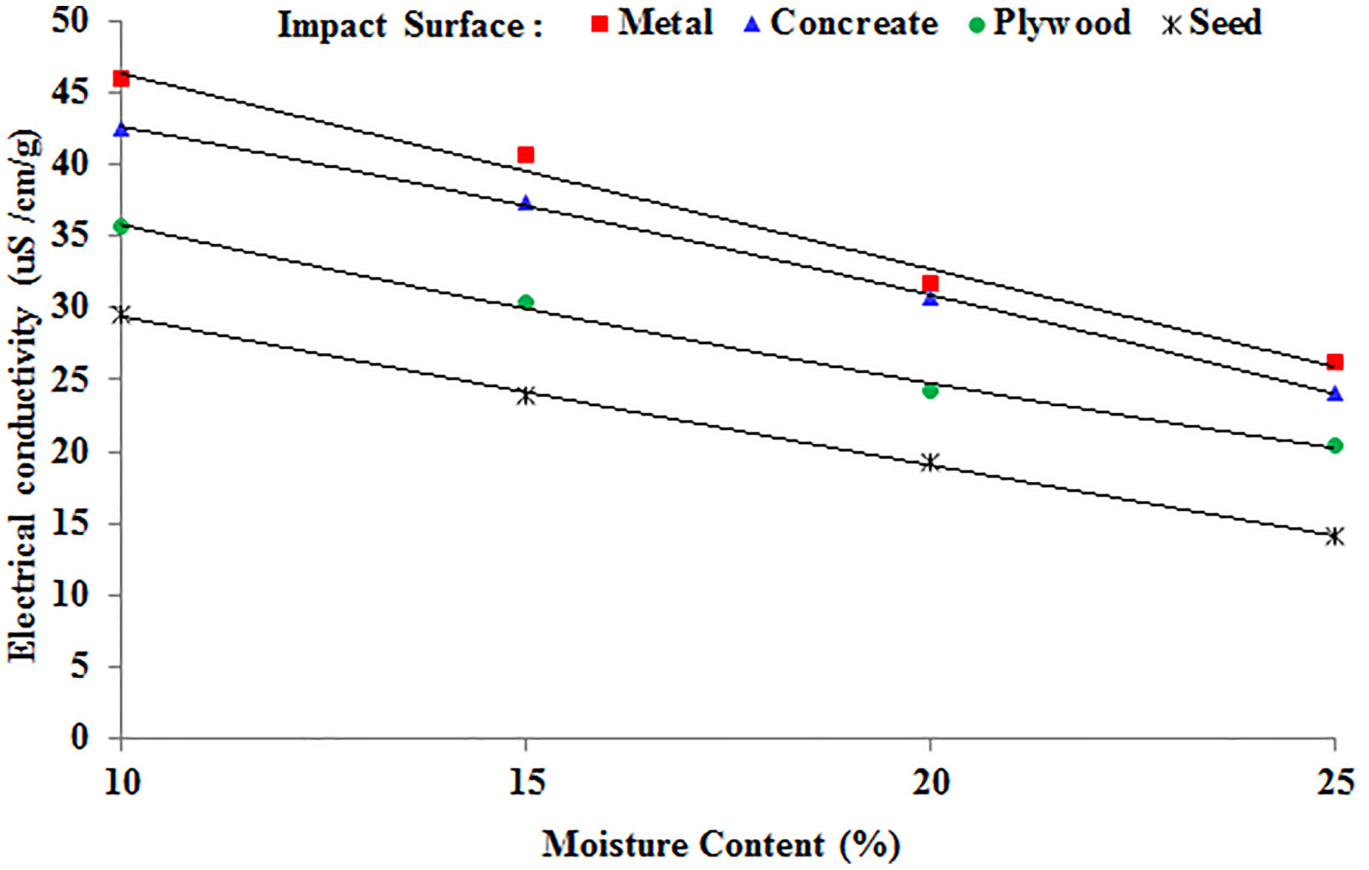
Change in the electrical conductivity of seeds in the interaction effect of moisture content and impact surface.

Figure 3 illustrates the interaction effect of impact surface and moisture content on the electrical conductivity of chickpea seeds. The average electrical conductivity values of chickpea seeds at the four moisture levels showed significantly lower differences when dropped on seeds compared to metal or concrete surfaces. Mid‐range damage data were collected from seeds dropped on plywood. A reduction in moisture content led to heightened damage for seeds in free fall on all impact surfaces. Nevertheless, the impact of moisture level was comparatively minor when seeds were dropped onto other seeds. The lowest average electrical conductivity value was 14.05 μS cm^−1^ g^−1^, which occurred when seeds interacted with each other at 25% moisture. The highest average electrical conductivity value was 94.45 μS cm^−1^ g^−1^, which was observed when the metal impact surface interacted with 10% moisture content.

### 3.5 Interaction effect of the drop height and impact surface

**FIGURE 4:**
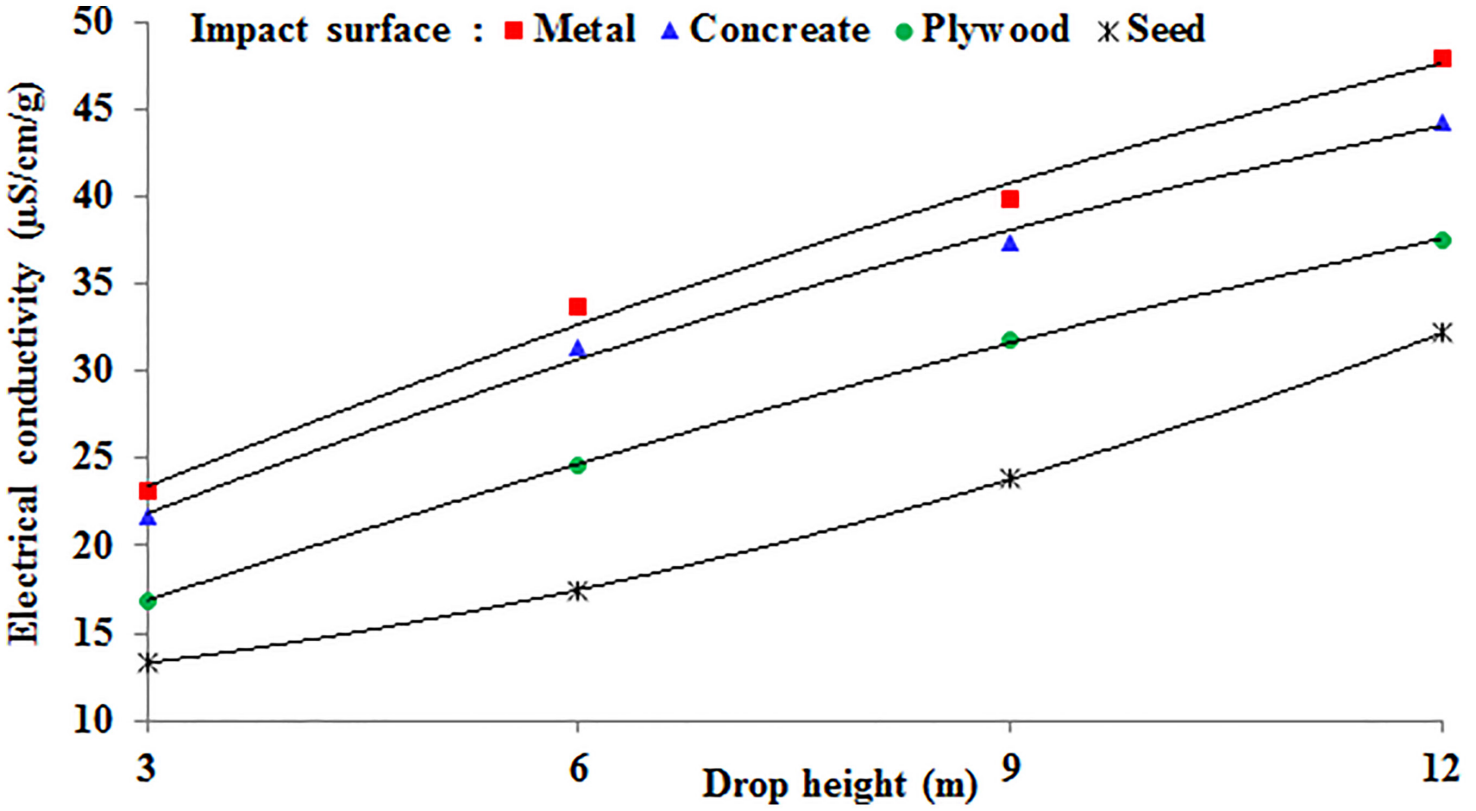
Variation of the electrical conductivity of chickpea seeds in the interaction effect of impact surface and drop height.

Figure 4 illustrates how the drop height and impact surface interaction influence the level of internal damage to chickpea seeds, as indicated by a change in electrical conductivity. The seeds exhibited a significantly greater change in electrical conductivity when dropped onto metal or concrete surfaces. Moreover, this difference was more pronounced at a drop height of 12 m compared to drop heights of 9, 6, and 3 m.

### 3.6 Interaction effect of moisture content and drop height

**FIGURE 5:**
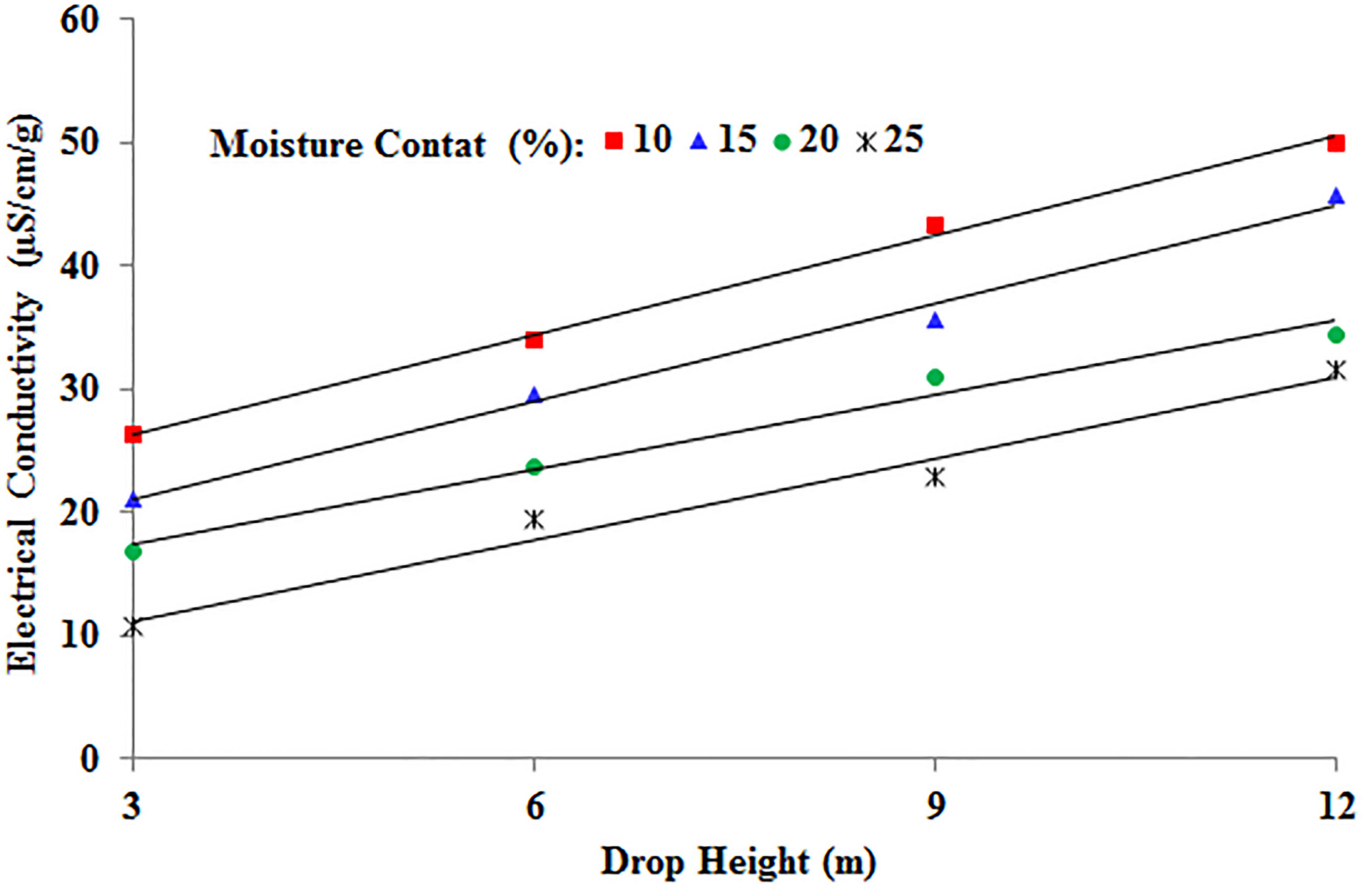
Change in the electrical conductivity of chickpea seeds in the interaction effect of drop height and moisture content.

Shown in Figure 5 is the interaction effect of the moisture content and drop height on the change in the electrical conductivity of chickpea seeds. The electrical conductivity of chickpea seeds increased as the drop height increased and as the moisture content decreased. The interaction of a drop height of 3 m and a moisture content of 25% resulted in the lowest mean value of electrical conductivity at 10 μS cm^−1^ g^−1^. Conversely, the interaction of a moisture content of 10% and drop height of 12 m produced the highest mean value of electrical conductivity at 50.06 μS cm^−1^ g^−1^. Therefore, to avoid internal cracking, the impact on the seeds should be low in intensity and energy.

### 3.7 Velocity of chickpea seeds during the free fall

Table 3 presents the average velocities of chickpea seeds dropped from varying heights, including both mass flow and individual seed velocities. The data in Table 3 indicate that the mass velocity values were higher compared to the velocities of individually dropped seeds at different drop heights due to air resistance in the drop tubes. Furthermore, the data in Table 3 show a significant increase in the velocity of the mass flow of chickpea seeds as the drop height increased. Specifically, the average mass flow velocities of chickpeas at drop heights of 3, 6, 9, and 12 m were 5.25, 7.35, 9.17, and 11.95 m s^−1^, respectively. Correspondingly, the mean values of germination loss at those velocities were 27.74%, 32.52%, 39.08%, and 48.08%, respectively. Additionally, the mean values of electrical conductivity of the seeds at those velocities were 18.72, 26.73, 33.16, and 40.47 μS cm^−1^ g^−1^, respectively. These findings indicate that as the drop height increased, the impact velocity on the seeds also increased, increasing both types of damage: a decrease in germination percentage and an increase in electrical conductivity. Moreover, when chickpea seeds were free‐fallen from heights greater than 6 m, the impact velocities exceeded 7 m s^−1^, causing the germination percentage and electrical conductivity values of the seeds to deviate from normal states. It has been reported that seed damage increases with higher impact velocities, which is consistent with previous studies on chickpeas (Shahbazi, 2011). Similarly, Shahbazi et al. (2012) found that increasing the impact velocity from 5.5 to 15.0 m s^−1^ resulted in an increase in damage from 0.4% to 37.3% in average values when working with impact speed in bean grain.

**TABLE 3:** Average chickpea seed velocities measured by dropping seeds from various heights, including mass flow and single seed velocities.

Drop height (m)	Velocity (mass flow) (m s^−1^)	Velocity (single seed) (m s^−1^)
3	5.25	8.83
6	7.35	8.02
9	9.17	10.12
12	11.95	13.03

Overall, this study underscores the importance of considering drop height, impact surface, and seed moisture content when assessing the physiological damage to seeds. Based on the results of this study, to reduce the mechanical damage to chickpea seeds, various strategies can be implemented throughout the supply chain. Firstly, proper harvesting techniques should be employed to minimize mechanical damage to seeds. This includes using appropriate harvesting equipment and ensuring that the seeds are not dropped from excessive heights. During handling and transportation, it is important to use gentle handling methods and avoid rough surfaces that can cause impact stress on seeds. Packaging materials should also be chosen carefully to provide cushioning and protection for the seeds.

It is worth noting that the experimental study of simulating free‐fall damage to seeds has certain limitations that can be investigated in future research. One of the limitations is the impact of PVC pipes on the free fall conditions of seeds. Here, the authors assumed that such an effect is negligible. However, dedicated research is needed to confirm such a hypothesis and quantify the potential impact of PVC pipes on free‐fall simulation. Another limitation is the accurate simulation of the free fall of the seeds in the air. While the authors tried to minimize differences in the velocity and height of the vertical fall of seeds, variations may exist between controlled laboratory conditions and unconstrained free fall scenarios. Therefore, studying the difference in seed behavior between free fall in air and a confined tube is another intriguing topic for future research. Additionally, this study only examined the impact of a single temperature (20°C). However, future research should investigate the effects of low temperatures on chickpea seed damage during free fall, which is crucial for simulating the loading and unloading of grain silos under different environmental temperatures.

A potential topic to consider in future research is exploring the correlation between seed damage, particularly in terms of germination percentage and microstructural data obtained from X‐rays (Nadimi et al., 2022; Nadimi, Divyanth, & Paliwal, 2023). Furthermore, advanced machine vision tools like imaging or spectroscopy could be utilized to automate the assessment of mechanical damage in seeds (Hosainpour et al., 2022; Nadimi et al., 2021; Nadimi, Divyanth, Chaudhry, et al., 2023; Nadimi, Hawley, Liu, et al., 2023). It is worth noting that the current study only focused on damage to chickpea seeds. To enhance the generalizability of these findings, it would be beneficial to extend this research to other legumes, grains, and oil seeds.

## 4 CONCLUSION

The study found that impact damage caused by free fall had significant negative effects on the quality of chickpea seeds. This damage led to seed deterioration, as seen in the decrease in germination percentage during the accelerated aging test, as well as the increase in electrical conductivity. Several factors influenced the extent of seed damage, including drop height, impact surface type, and seed moisture. Notably, dropping the chickpea seeds on metal or concrete surfaces resulted in significantly higher damage compared to plywood and seed‐to‐seed. Increasing the drop height also increased the damage inflicted on the seeds. The maximum internal damage occurred when the chickpea seeds struck a surface at speeds exceeding 7 m s^−1^. To minimize impact damage to chickpea seeds from free fall, it is recommended to limit the drop height to approximately 6 m. Chickpea seed moisture content emerged as a significant factor influencing seed deterioration due to impact damage. Seeds with higher moisture content experienced greater physiological damage, as moisture can facilitate the degradation of seed tissues and accelerate the aging process. Conversely, seeds with lower moisture content exhibited relatively higher levels of electrical conductivity, indicating the importance of maintaining optimal moisture levels for seed preservation.

## AUTHOR CONTRIBUTIONS

**Farzad Delfan:** Data curation (equal); methodology (equal). **Feizollah Shahbazi:** Data curation (equal); investigation (equal); software (equal); writing – original draft (equal). **Hamid Reza Eisvand:** Data curation (equal); visualization (equal). **Saba Shahbazi:** Writing – review and editing (equal).

## FUNDING INFORMATION

This research received no funding.

## CONFLICT OF INTEREST STATEMENT

The authors declare that they have no competing interests.

## ETHICAL APPROVAL

This study does not involve humans or animals.

## Data Availability

Research data are not shared.
